# Risk Factors for Foodborne Zoonoses Among Populations With and Without a Migration Background in Berlin, Germany

**DOI:** 10.3390/tropicalmed10100281

**Published:** 2025-09-30

**Authors:** Idesbald Boone, Sabrina Janßen, Tanguy Marcotty, Verena Moos, Kristina Allers, Anika Geelhaar-Karsch, Thomas Schneider, Sascha Al Dahouk

**Affiliations:** 1 Department of Biological Safety, German Federal Institute for Risk Assessment, Diedersdorfer Weg 1, 12277 Berlin, Germany; 2 Division of Gastroenterology, Infectious Diseases and Rheumatology, Campus Benjamin Franklin, Charité–Universitätsmedizin Berlin, Corporate Member of Freie Universität Berlin and Humboldt-Universität zu Berlin, Hindenburgdamm 30, 12203 Berlin, Germany; 3 Department of Veterinary Medicine, Faculty of Sciences, University of Namur (UNamur), Rue de Bruxelles 61, 5000 Namur, Belgium; 4 Department of Internal Medicine, RWTH Aachen University Hospital, Pauwelsstraße 30, 52074 Aachen, Germany

**Keywords:** foodborne zoonoses, seroprevalence, risk factors, migrant population, Berlin, Germany

## Abstract

Knowledge gaps exist regarding foodborne zoonotic diseases in migrant populations. We assessed the seroprevalence of *Campylobacter*, *Salmonella*, *Yersinia*, *Brucella*, hepatitis E virus (HEV), and *Trichinella*, and identified potential exposure risks in populations with and without migration backgrounds. In a cross-sectional study (2014–2016), adults with Turkish, Russian, Vietnamese, or German backgrounds residing in Berlin, Germany, were recruited via convenience sampling. Sera were screened for anti-IgG antibodies, and risk factors were assessed via a structured questionnaire. Logistic regression was used for analysis. We included 1180 participants: 497 Germans and 215, 273, and 195 individuals with Russian, Turkish, and Vietnamese backgrounds, respectively. *Salmonella* seroprevalence was highest among Vietnamese (47–50%) and lowest among Turks (18–20%). Campylobacter seroprevalence ranged from 17% to 23%. *Yersinia* seroprevalence was highest among Germans (64–70%) and associated with raw pork consumption. HEV seropositivity was highest among Vietnamese (27–28%) and lowest among Russians (5%). No samples were positive for *Brucella*; two were positive for *Trichinella*. High seroprevalence of *Campylobacter*, *Salmonella*, *Yersinia*, and HEV suggests substantial exposure and frequent asymptomatic or mild infections. While *Yersinia* seropositivity was associated with raw pork consumption, high seroprevalence in Turks—who rarely consume pork—suggests other food sources or transmission routes.

## 1. Introduction

Foodborne zoonoses (FBZ) are infectious diseases that are naturally transmitted from vertebrate animals to humans through the consumption of contaminated food, mainly meat products and eggs. They can cause considerable morbidity and mortality [1], as well as economic losses worldwide.

Similar to trends in the European Union (EU) [2], the highest incidences of bacterial zoonoses in Germany in 2016 (the notification year corresponding with our study period, 2014–2016) were reported for campylobacteriosis, salmonellosis, and yersiniosis, with 89.8, 15.7, and 3.4 cases per 100,000 population, respectively [3]. These incidences decreased to 47.7, 12.7, and 2.3 in 2023. However, because of a lack of awareness and underdiagnosis, the actual incidences are likely higher. Other FBZ of public health relevance in Germany include hepatitis E virus (HEV), which has been increasingly reported (rising from 1994 cases in 2016 to 4658 in 2023), brucellosis (with 36 cases in both 2016 and 2023), and trichinellosis (four cases in 2016 and three in 2023) [3]. Fever and gastrointestinal symptoms are common in patients with foodborne diseases; however, more specific clinical signs may also occur, such as undulant fever and sacroiliitis in patients with brucellosis, periorbital oedema in those with trichinellosis, and jaundice in those with hepatitis E. While most acute infections are self-limiting, FBZ can lead to severe chronic sequelae, including reactive arthritis, irritable bowel syndrome, and Guillain–Barré syndrome [4,5].

The major factors influencing the epidemiology of foodborne diseases include demographics, global food production and markets, the increasing international trade of animals, global supply chains of meat and meat products, tourism, and consumption habits (e.g., consumption of raw or undercooked meat) [6]. Furthermore, climate change is expected to contribute to a rise in foodborne infections and intoxications [7].

Berlin is a multicultural city with approximately 3.7 million inhabitants, of whom one-third (32%) have a migration background [8]. In 2016, the largest non-EU migrant communities in Berlin included the Turkish (176,730 individuals), Russian (53,753), and Vietnamese (27,637) populations. Data on the exposure of migrant populations to zoonotic foodborne pathogens are lacking despite potential differences in food consumption habits and travel behaviour. Migrant populations are often underrepresented in national health surveys because of language barriers, sociocultural factors, or difficulties in reachability. In Germany, migration background is not routinely recorded for FBZ but is documented for a selection of infectious diseases (e.g., tuberculosis and human immunodeficiency virus). Moreover, different indicators for migration background are applied, such as nationality, country of birth, and country of origin [9].

Serological data on FBZ in the general population of Germany are scarce and primarily limited to seroprevalence studies on HEV and *Brucella*, while data on *Campylobacter*, *Salmonella*, *Yersinia*, or *Trichinella* are lacking. Representative seroprevalence and exposure data for migrant populations in Germany are even more limited or entirely absent, with information, at best, available for their countries of origin.

This study had two primary aims. First, we estimated the seroprevalence of anti-*Campylobacter*, anti-*Salmonella*, anti-*Yersinia*, anti-*Brucella*, anti-HEV, and anti-*Trichinella* antibodies in populations without a migration background (Germans) and in those with a Russian, Turkish, or Vietnamese background because these groups represent the largest non-EU populations in Berlin. Second, we identified factors associated with seropositivity because combining population-specific seroprevalence data with potential risk factors can support targeted public health prevention and control measures to better protect at-risk communities.

## 2. Materials and Methods

### 2.1. Study Population, Questionnaire, and Blood Sampling

We used a cross-sectional survey design, restricting participation to healthy adults (aged ≥ 18 years) with a Russian, Turkish, or Vietnamese background or Germans (i.e., without a migration background) residing in Berlin or Brandenburg. All participants were recruited between February 2014 and November 2016. Migration background was defined by foreign nationality, personal migration history, or having at least one parent who migrated to Germany. First-generation migrants were individuals who had experienced migration themselves and were not born in Germany, while second-generation migrants were those born in Germany to parents who were not (i.e., children of first-generation migrants) [10]. A convenience sampling approach was chosen as the most feasible method to reach migrant communities. Initially, sampling sites included wards in a 1030-bed university hospital in southwest Berlin (Charité—Universitätsmedizin Berlin, Campus Benjamin Franklin), excluding those treating infectious diseases. Based on prevalence estimates from the literature [11,12,13,14], we calculated a sample size of 500 participants per subpopulation to allow for statistical detection of frequency differences using chi-squared (χ^2^) tests with a power of at least 80% and a significance level of α = 0.05. Sample size calculations were performed using nQuery 6.0 (Statistical Solutions Ltd., Boston, MA, USA).

Because the intended sample size per subpopulation could not be achieved for migrants by including only patients and visitors, we expanded the sampling sites to outpatient clinics with a high number of migrant patients, immigrant associations, ethnic shops and restaurants, and migrant-focused events. Recruitment was carried out mainly in Berlin districts with higher concentrations of specific subpopulations. To facilitate recruitment, we consulted stakeholders from migrant associations, and if the chairperson expressed interest in our study, we organised informative group meetings on foodborne zoonotic infections and preventive measures in the participants’ preferred languages (German, Russian, Turkish, or Vietnamese). The field team included at least a study nurse and interviewers with the necessary language skills. Participants who provided written informed consent, met the inclusion criteria, completed a structured questionnaire, and provided a blood sample were included in this study. The questionnaire covered demographic characteristics (age, sex, household size, migrant background, level of professional education), travel history, exposure to animals, food handling, food consumption habits, risk awareness, and medical history. Data entry and quality control were carried out by aproxima (Gesellschaft für Markt- und Sozialforschung Weimar mbH, Thuringia, Germany) using “Command Center” from VOXCO (Voxco GmbH, Mannheim, Germany).

Whole blood samples (approximately 20 mL) were collected using Vacutainer tubes (BD Vacutainer SSTm Advance Tube; Becton Dickinson, Franklin Lakes, NJ, USA). After collection, the samples were allowed to clot at room temperature for 30 min. Serum was then transported to the laboratory on ice, separated by centrifugation at 2000× *g* for 10 min, and stored at −80 °C until use.

### 2.2. Serological Testing

Commercial test kits were used for antibody detection according to the manufacturers’ instructions, with all serum samples tested in duplicate. We screened serum samples for *Salmonella* IgG/IgA/IgM antibodies using enzyme-linked immunosorbent assay (ELISA) (IMTEC-*Salmonella*-Antibodies Screen; AlphaScience GmbH, Riedstadt, Germany), confirming positive and inconclusive results with a second ELISA (IMTEC-*Salmonella*-Antibodies IgA; AlphaScience GmbH). Serum samples were also tested for anti-*Campylobacter* IgG using a strip-immunoassay (recomLine *Campylobacter* IgG; Mikrogen GmbH, Neuried, Germany) and for anti-*Yersinia* IgG using another strip-immunoassay (recomLine *Yersinia* IgG; Mikrogen GmbH). Additionally, we screened for anti-HEV IgG with ELISA (recomWell HEV IgG; Mikrogen GmbH), confirming positive or inconclusive results with a strip-immunoassay (recomLine HEV IgG; Mikrogen GmbH). Further testing included anti-*Brucella* IgG by ELISA (*Brucella* IgG kit; Virion/Serion, Würzburg, Germany), with confirmation via in-house serum agglutination and complement fixation tests. Anti-*Trichinella* IgG antibodies were measured using ELISA (*Trichinella spiralis* IgG ELISA; DRG Instruments GmbH, Marburg, Germany), with positive results confirmed by an in-house Western blot (IgG and IgM) [15].

### 2.3. Data Analysis

We calculated the seroprevalence (percentage of positively tested individuals) and 95% confidence intervals (CIs) for anti-*Campylobacter*, *Salmonella*, *Yersinia*, *Brucella*, HEV, and *Trichinella* antibodies in the four subpopulations (Germans, Russians, Turks, and Vietnamese) and aggregated frequency scales of categorical variables with very low response rates (<10). Univariable logistic regression was used to identify associations between potential exposure variables (explanatory variables) and seropositivity (response variable), with explanatory variables including migration background, migrant generation, age at migration, sex, age, self-assessed German language skills, household size, level of professional education, travel history within the last 3 years, receiving guests from abroad, exposure to animals (pets, livestock, wildlife) and frequency of exposure, professional exposure to animal products (e.g., meat industry, restaurants, shops, hunting, farming), origin of meat product purchases (supermarket, retail, market, internet, directly from the producer), importing animal food products from abroad, dietary habits, consumption and frequency of products (meat, meat products, fish, non-pasteurised milk), preparation of meat products or fish at home, handling of frozen meat products and thawing methods, cautiousness when buying/eating animal products because of potential illness risk, alcohol consumption, smoking, chronic diseases, and common symptoms associated with foodborne infections. The multivariable logistic regressions included explanatory variables with a *p*-value of <0.05 from the univariable logistic analysis, followed by backwards elimination of insignificant variables, with the likelihood ratio test used to select the most simplified model. Post-estimation of the final model included a goodness-of-fit test and a link test to assess model fit and specification error. To ensure seroprevalence comparability between subpopulations independent of age effects, seroprevalence estimates were presented for an average individual aged 40–49 years. A multivariable logistic regression analysis including sex, age group, and subpopulation as explanatory variables was performed. All statistical analyses were conducted using STATA 17 (StataCorp, College Station, TX, USA), with *p*-values of <0.05 considered significant.

## 3. Results

### 3.1. Descriptive Analysis

In total, 1180 participants met the inclusion criteria, with the majority (n = 497, 42%) of German origin (i.e., without a migration history), while 215 (18%), 273 (23%), and 195 (17%) participants had a Russian, Turkish, and Vietnamese background, respectively. Of these, 93% were residents of Berlin, and 7% came from neighbouring districts in the federal state of Brandenburg. Participants of German origin were primarily recruited from among patients and visitors at Charité, Campus Benjamin Franklin, and the Occupational Health Centre of Charité (n = 407, 82%), whereas those with a Russian, Turkish, or Vietnamese background were mainly recruited through migrant association clubhouses (Table 1).

Demographic characteristics, including age, sex, migrant generation, and age at migration for the four subpopulations, are presented in Table 2. In total, 25% of Germans, 28% of Turks, 17% of Russians, and 19% of Vietnamese participants reported privately importing animal food products from abroad to Germany at least once. Turkish and Vietnamese participants primarily brought these products from their home countries (100% for Turks and 82% for Vietnamese), whereas only 54% of imports reported by Russians originated from Russia, with the remainder coming from various countries, mostly within the EU/European Economic Area (EEA). By contrast, 96% of the imports reported by Germans originated from EU/EEA countries.

### 3.2. Seroprevalence

**Figure 1:**
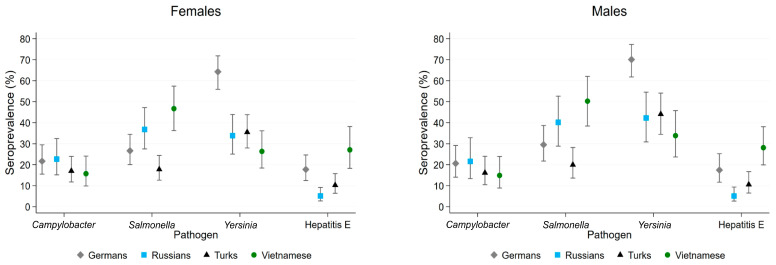
Seroprevalence of anti-*Campylobacter* IgG, anti-*Salmonella* IgA, anti-*Yersinia* IgG, and anti-hepatitis E virus IgG in female (**left**) and male (**right**) participants aged 40–49 years, by subpopulation group, sampled in Berlin from 2014 to 2016. Error bars represent 95% confidence intervals. Seroprevalence for *Trichinella* and *Brucella* is not shown.

The percentage of participants with positive anti-*Campylobacter* IgG, anti-*Salmonella* IgG/IgA/IgM, anti-*Yersinia* IgG, and anti-HEV IgG in the four subpopulations is shown by sex in Figure 1. No study participant tested positive for anti-*Brucella* IgG, while two Vietnamese participants (a 38-year-old man and a 27-year-old woman) tested positive for anti-*Trichinella* IgG or IgM. *Campylobacter* seropositivity was similar among Turkish and Vietnamese men and women, at 17% and 16%, respectively, whereas within the Russian subpopulation, 23% of women and 22% of men were seropositive. The prevalence of antibodies to *Salmonella* ranged from 18% in Turkish women and 20% in Turkish men to 47% in Vietnamese women and 50% in Vietnamese men. The highest *Yersinia* seropositivity was observed among Germans, with 64% in women and 70% in men, while lower rates were found in the Russian (34% in women, 42% in men), Turkish (36% in women, 44% in men), and Vietnamese subgroups (26% in women, 34% in men). In total, 137 of 503 (27%) *Yersinia*-seropositive serum samples could be attributed to *Y. enterocolitica* (n = 73, 53%) and *Y. pseudotuberculosis* (n = 64, 47%) based on the pattern of *Yersinia* outer proteins detected by anti-*Yersinia* IgG antibodies. The highest HEV seropositivity was found in Vietnamese participants (27–28%), followed by Germans (17–18%), Turks (10–11%), and Russians (5%).

**Figure 2:**
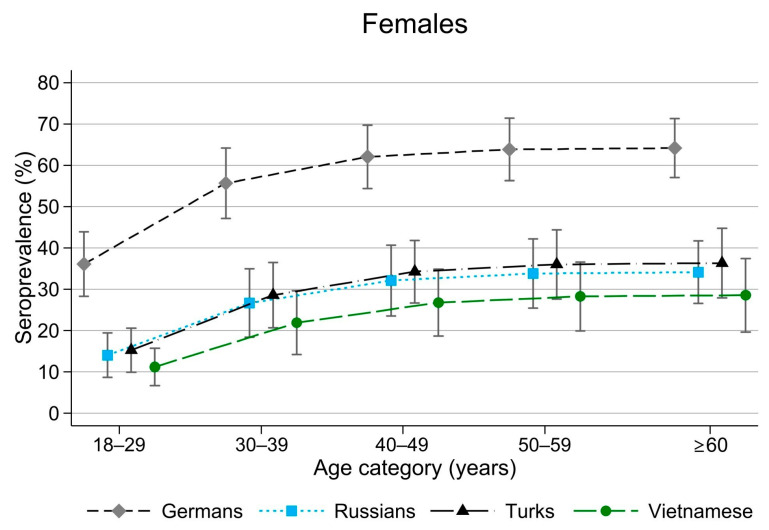
Seroprevalence of anti-*Yersinia* IgG by age in female participants across the four subpopulation groups sampled in Berlin from 2014 to 2016.

*Yersinia* seroprevalence increased with age (see Figure 2, which presents data only from women because results for men were qualitatively similar). This increase was significant in the German subpopulation, rising from 36% (95% CI: 28–44%) in the 18- to 29-year age category to 64% (95% CI: 57–71%) in those aged ≥60 years.

**Figure 3:**
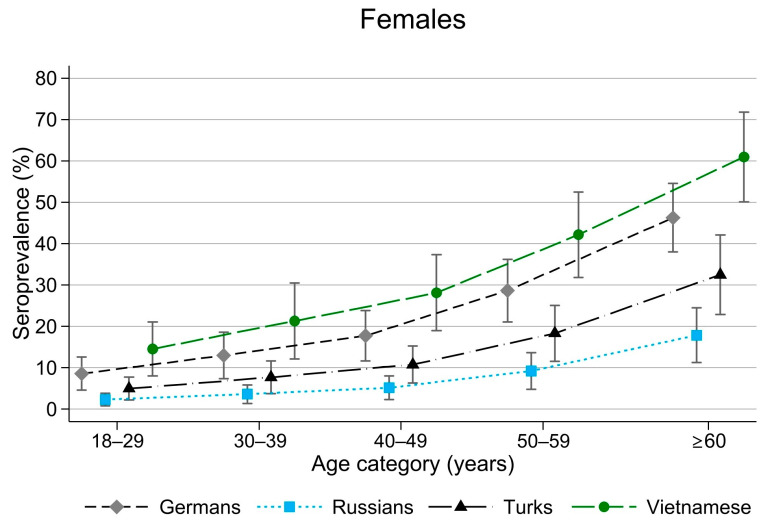
Seroprevalence of anti-hepatitis E virus IgG by age in female participants across the four subpopulation groups sampled in Berlin from 2014 to 2016.

Age dependency was also observed for HEV seropositivity (see Figure 3, which presents data only from women because results for men were qualitatively similar). In the German subpopulation, HEV seropositivity increased from 9% (95% CI: 5–13%) in the 18- to 29-year age group to 46% (95% CI: 40–54%) in those aged ≥60 years. In the Turkish subpopulation, it ranged from 5% (95% CI: 2–8%) in the 18- to 29-year age group to 32% (95% CI: 23–42%) in those aged ≥60 years. Among Vietnamese participants, seropositivity increased from 15% (95% CI: 8–22%) in the 18- to 29-year age group to 61% (95% CI: 50–72%) in those aged ≥60 years. No age dependency was observed for *Salmonella* seropositivity.

Overall, a higher anti-*Campylobacter* IgG seroprevalence was observed in the youngest age group (18–29 years). Among German women, the largest difference in seropositivity was observed between participants aged 18–29 years (32%, 95% CI: 25–40%) and those aged 50–59 years (15%, 95% CI: 10–21%).

### 3.3. Association Between Seropositivity and Potential Risk Factors

Risk factor analysis was conducted to identify variables associated with *Campylobacter*, *Salmonella*, *Yersinia*, and HEV seropositivity among all study participants (Table 3) as well as within each subpopulation. Risk factors related to *Brucella* (all negative) and *Trichinella* serological status were not analysed because of the low seropositivity. Odds ratios (ORs) are provided in the following sections to improve clarity and readability.

#### 3.3.1. Campylobacter

The univariable analysis identified several factors significantly associated with *Campylobacter* seropositivity, including direct contact with horses (OR: 2.3, 95% CI: 1.2–4.3) or rabbits (OR: 1.8, 95% CI: 1.1–2.9) and the consumption of game (OR: 1.4, 95% CI: 1.04–1.9), raw pork sausages (OR: 1.4, 95% CI: 1.01–1.8), raw beef (OR: 1.4, 95% CI: 1.04–1.9), beef ham (OR: 1.4, 95% CI: 1.01–1.8), turkey (OR: 1.4, 95% CI: 1.01–1.9), seafood (OR: 1.4, 95% CI: 1.1–1.9), caviar (OR: 1.4, 95% CI: 1.02–1.9), smoked fish (OR: 1.4, 95% CI: 1.04–1.9), and raw fish (OR: 1.6, 95% CI: 1.2–2.2). Older age was negatively associated with seropositivity (see Section 3.2), and in a multivariable logistic regression adjusted for age, raw fish consumption remained statistically significantly associated with seropositivity (Table 3).

In the German subpopulation, living in a household with more than one person (OR: 2.1, 95% CI: 1.3–3.6) and purchasing food from Turkish grocery stores (OR: 1.8, 95% CI: 1.2–2.8) were positively associated with *Campylobacter* seropositivity. In the Turkish subpopulation, second-generation migration (OR: 2.8, 95% CI: 1.5–5.6), receiving guests from abroad (OR: 2.1, 95% CI: 1.1–4.0), and the consumption of pork (OR: 3.0, 95% CI: 1.1–8.1), turkey (OR: 2.3, 95% CI: 1.1–5.0), raw fish (OR: 2.2, 95% CI: 1.1–4.5), and shopping in Asian grocery stores (OR: 3.6, 95% CI: 1.2–10.8) were significantly associated with *Campylobacter* seropositivity in the univariable logistic regression. No significant associations were found in the logistic regression analysis for the Russian subpopulation. In the Vietnamese subpopulation, second-generation migration (OR: 6.8, 95% CI: 1.7–26.7), consumption of ham (OR: 2.4, 95% CI: 1.1–5.1), beef sausage (OR: 2.4, 95% CI: 1.1–5.2), raw fish (OR: 3.0, 95% CI: 1.4–6.6), smoked fish (OR: 3.3, 95% CI: 1.5–7.2), and caviar (OR: 3.1, 95% CI: 1.4–6.6) were positively associated with *Campylobacter* seropositivity.

#### 3.3.2. Salmonella

In the univariable analysis including all participants, *Salmonella* seropositivity was significantly positively associated with purchasing animal food products from Asian supermarkets (OR: 1.6, 95% CI: 1.2–2.1), following a vegetarian diet (OR: 1.7, 95% CI: 1.1–2.6) or an unrestricted diet (OR: 1.7, 95% CI: 1.03–1.8), as well as consuming pork (OR: 1.6, 95% CI: 1.2–2.2) and seafood (OR: 1.4, 95% CI: 1.1–1.8). By contrast, eating halal food (OR: 0.5, 95% CI: 0.3–0.7), consuming chicken (OR: 0.5, 95% CI: 0.3–0.7), turkey (OR: 0.5, 95% CI: 0.3–0.8), sheep (OR: 0.6, 95% CI: 0.5–0.8), or beef (OR: 0.5, 95% CI: 0.3–0.9); purchasing meat products directly from farms (OR: 0.6, 95% CI: 0.4–0.8); and careful handling of animal food products (OR: 0.7, 95% CI: 0.6–0.9) were negatively associated with *Salmonella* seropositivity.

Eating chicken meat remained negatively associated with seropositivity in the multivariable model adjusted for sex and migration background (Table 3).

In the multivariable logistic regression restricted to the German subpopulation, male sex (OR: 1.8, 95% CI: 1.2–2.7) and having imported animal food products (OR: 1.6, 95% CI: 1.03–2.5) were statistically positively associated with *Salmonella* seropositivity.

Among Russians, seropositive individuals were more likely to have limited German language skills (OR: 2.4, 95% CI: 1.3–4.3). In the Vietnamese subpopulation, a vegetarian lifestyle (OR: 4.9, 95% CI: 1.7–13.8) was significantly associated with *Salmonella* seropositivity, while in the Turkish subpopulation, consumption of chicken (OR: 0.2, 95% CI: 0.1–0.6) was significantly negatively associated in the univariable analysis.

#### 3.3.3. Yersinia

German origin (OR: 3.3, 95% CI: 2.5–4.2), male sex (OR: 1.6, 95% CI: 1.3–2.1), and age (compared with 18–29 years: OR: 2.1, 95% CI: 1.4–3.2 for 30–39 years; OR: 2.2, 95% CI: 1.5–3.2 for 40–49 years; OR: 2.5, 95% CI: 1.7–3.7 for 50–59 years; OR: 2.8, 95% CI: 1.9–4.1 for ≥60 years), living in a single household (OR: 1.4, 95% CI: 1.04–1.8), not having travelled abroad (OR: 1.5, 95% CI: 1.2–2.0), contact with cats (OR: 1.4, 95% CI: 1.1–1.9), contact with dogs (OR: 1.6, 95% CI: 1.3–2.1), contact with mice (OR: 1.8, 95% CI: 1.1–3.0), buying meat products from German retail shops (OR: 2.6, 95% CI: 2.0–3.3), buying meat products directly from farms (OR: 1.8, 95% CI: 1.3–2.4), a diet without restrictions (OR: 1.4, 95% CI: 1.1–1.8), and consumption of pork (OR: 1.8, 95% CI: 1.3–2.4), especially raw pork (minced raw pork called ‘Hackepeter’ or ‘Schweinemett’ in Germany and the traditional raw pork sausage ‘Mettwurst’), as well as beef (OR: 1.7, 95% CI: 1.2–2.5), poultry (OR: 1.7, 95% CI: 1.2–2.5), game (OR: 2.7, 95% CI: 2.1–3.6), horsemeat (OR: 3.8, 95% CI: 2.0–7.2), and raw milk (OR: 1.5, 95% CI: 1.2–2.0), were positively associated with *Yersinia* seropositivity in the univariable analysis including all participants. Halal food (including the prohibition of pork consumption) (OR: 0.6, 95% CI: 0.5–0.9) and careful handling of animal food products (OR: 0.6, 95% CI: 0.5–0.8) were statistically negatively associated with *Yersinia* seropositivity.

The consumption of raw pork, German origin (absence of migration background), and purchasing meat products from German retail shops remained positively associated with *Yersinia* seropositivity in the multivariable logistic regression, while careful handling of animal food products remained negatively associated (Table 3). The association with raw pork consumption was stronger with increased frequency (OR: 3.4, 95% CI: 1.6–7.2).

In the multivariable model restricted to the German subpopulation, age, frequent raw pork consumption (OR: 8.0, 95% CI: 2.2–28.9), and careful handling of animal food products (OR: 0.6, 95% CI: 0.4–0.9) remained statistically significantly associated with *Yersinia* seropositivity. By contrast, in the multivariable model restricted to the Vietnamese subpopulation, male sex (OR: 3.6, 95% CI: 1.6–8.0) and living in a single household (OR: 2.7, 95% CI: 1.1–6.8), after adjusting for age, were significantly positively associated with *Yersinia* seropositivity, while a diet without restrictions was negatively associated (OR: 0.3, 95% CI: 0.1–0.8). No variables were found to be associated with *Yersinia* seropositivity in the Russian and Turkish subpopulations.

#### 3.3.4. HEV

In the univariable analysis, increasing age was statistically positively associated with HEV seropositivity (compared with 18–29 years: OR: 1.3, 95% CI: 0.7–2.5 for 30–39 years; OR: 1.9, 95% CI: 1.03–3.3 for 40–49 years; OR: 3.4, 95% CI: 2.0–5.8 for 50–59 years; OR: 5.6, 95% CI: 3.3–9.4 for ≥60 years), along with purchasing meat products from organic shops (OR: 1.9, 95% CI: 1.2–3.1) and consuming horsemeat (OR: 2.0, 95% CI: 1.1–3.8). By contrast, HEV seropositivity was statistically negatively associated with travelling in the last 3 years (OR: 0.6, 95% CI: 0.5–0.9), purchasing meat products from Russian (OR: 0.5, 95% CI: 0.3–0.8) or Turkish supermarkets (OR: 0.7, 95% CI: 0.5–0.9), consuming halal food (OR: 0.6, 95% CI: 0.4–0.9), and eating raw fish (OR: 0.6, 95% CI: 0.4–0.8). Compared with the Vietnamese subpopulation, participants from the Russian (OR: 0.3, 95% CI: 0.2–0.5) and Turkish subpopulations (OR: 0.4, 95% CI: 0.2–0.6) had lower odds of HEV seropositivity.

In the multivariable model including all participants, age and migration background remained significantly associated with HEV seropositivity (Table 3).

In the univariable analysis restricted to the German subpopulation, increasing age was significantly associated with HEV seropositivity (compared with 18–29 years: OR: 0.9, 95% CI: 0.3–2.3 for 30–39 years; OR: 2.5, 95% CI: 1.7–5.8 for 40–49 years; OR: 4.3, 95% CI: 2.0–9.84 for 50–59 years; OR: 8.7, 95% CI: 4.1–18.1 for ≥60 years). In the Russian subpopulation, living in a household with more than one person was significantly associated with seropositivity (OR: 4.9, 95% CI: 1.4–17.2). In the Turkish subpopulation, consumption of turkey was positively associated with HEV seropositivity (OR: 4.4, 95% CI: 1.6–12.1) in a multivariable model adjusted for age.

## 4. Discussion

Our cross-sectional study estimated the seroprevalence of antibodies against six foodborne zoonotic pathogens (*Campylobacter*, *Salmonella*, *Yersinia*, HEV, *Brucella*, and *Trichinella*) in populations without a migration background (Germans) and those with a Russian, Turkish, or Vietnamese background in Berlin.

In Germany, only the notification of acute cases of campylobacteriosis, salmonellosis, yersiniosis (caused by *Y. enterocolitica* and *Y. pseudotuberculosis*), hepatitis E, brucellosis, and trichinellosis is mandatory [16]. Overall, our laboratory results indicated a high level of exposure to *Campylobacter*, *Salmonella*, *Yersinia*, and hepatitis E virus in the four subpopulations studied.

Compared with the seroprevalence (a mixture of IgG, IgM, and IgA) in the general population of the Netherlands, which was reported at 68.1% with an age-related increase reaching 100% in individuals older than 55 years [17], the *Campylobacter* seropositivity in the current study was considerably lower (15–23%) and did not increase with age. Similarly, a Danish study showed that IgG seroprevalence in the general population of Copenhagen increased from 20.6% in individuals aged 15–35 years to 32.4% in those aged 50–69 years [18]. By contrast, our multivariable analysis did not confirm previously described risk factors for *Campylobacter* exposure, such as the consumption of chicken [19], consumption of undercooked meat, increasing age, female sex, or having a migrant background [17]. The lack of association with chicken consumption was surprising, given that chicken is considered a major risk factor for *Campylobacter* infection. In our univariable analysis, only turkey consumption was associated with *Campylobacter* seropositivity. This may reflect differences in food preparation practices, as traditional cooking methods among Turkish and Vietnamese subpopulations often involve longer or more thorough cooking, thereby reducing the risk of exposure. Significant variables from the univariable analysis, such as the consumption of raw fish (sushi), which has been suggested as a possible vehicle for campylobacteriosis in Malaysia [20], require further investigation. Other routes of transmission, such as environmental exposure or contact with contaminated drinking or recreational water, may also play a role but were not covered in the present study.

The highest *Salmonella* seroprevalence was observed among Vietnamese and Russian study participants, while the lowest was found in the Turkish subpopulation, even when compared to individuals without a migration background. Although the incidence of salmonellosis in Berlin, with 515 cases notified in 2016, was reportedly six times lower than that of campylobacteriosis, the *Salmonella* seroprevalence in this study exceeded that of *Campylobacter*. There is a lack of comparative seroprevalence data from the native countries of the migrant populations. Interestingly, occasional or frequent consumption of chicken meat was associated with a reduced likelihood of *Salmonella* seropositivity in the multivariable analysis.

Despite the low incidence of yersiniosis (2.3 per 100,000 population) and HEV (3.2 per 100,000 population) reported in Berlin in 2016 [3], our study revealed a high seroprevalence, suggesting a significant number of asymptomatic or mild infections and/or underdiagnosis.

The seroprevalence of anti-*Yersinia* IgG was higher among German participants than in migrant populations. The lower *Yersinia* seropositivity in Turks than in Germans can be at least partly explained by lower pork consumption, as 93% of the Turkish subpopulation reported not eating pork. Notably, *Yersinia* seroprevalence was strongly associated with the consumption of raw pork products, such as ‘Schweinemett’ and ‘Hackepeter’, which are traditional foods frequently consumed by Germans but rarely reported among the migrant populations interviewed. The association between horsemeat consumption and *Yersinia* seroprevalence warrants further investigation because contamination of horsemeat has been reported to be relatively common [21].

The protective effect of careful handling of animal food products against *Yersinia* seropositivity suggests that raising awareness may help prevent yersiniosis. The still relatively high anti-*Yersinia* IgG seroprevalence in Turks (36–44%), as well as in Vietnamese (26–34%) and Russians (34–42%), indicates that other risk factors, such as salad consumption [22,23], might play a role but were not addressed by our questionnaire. Previous studies in Germany have reported a *Yersinia* seroprevalence of 30–40% among healthy blood donors [24,25]. In our study population, *Yersinia* seropositivity increased with age, similar to findings from a study on healthy Austrians [26], in which seroprevalence ranged from 24.7% in individuals aged 19–24 years to 38.5% in those aged 44–54 years (Figure 2).

The HEV seropositivity in the German subpopulation (17–18%) aligned with a representative population-based seroprevalence study among adults in Germany, which reported a prevalence of 15.3% [27]. In our study, HEV IgG seroprevalence was highest among Vietnamese participants (27–28%) and lowest among Russian participants (5%). Similarly, a high HEV IgG seroprevalence of 31.4% (95% CI: 22.5–40.9) was observed in Vietnam [28], whereas in the general population of the Russian Federation, it was 4.6% (95% CI: 4.4–4.8) between 2018 and 2020, although prevalence varied across regions and increased with age [29]. In Türkiye, the nationwide prevalence of total anti-HEV antibodies among blood donors ranged from 11.5% to 12.2% between 2014 and 2016 [30]. We initially hypothesised that the avoidance of pork consumption might explain the lower HEV seroprevalence in the Turkish population; however, this was not confirmed by our data. The consumption of offal (kidney, liver, gut) and wild boar meat [31], and occupational contact with pigs [32] have previously been associated with an increased HEV IgG seroprevalence. These specific food products were not explicitly addressed in our questionnaire, and the number of participants who had direct contact with pigs was negligible in this study. In Europe, the primary transmission route of HEV (typically genotype 3) is assumed to be foodborne, whereas in low-income countries, HEV (mainly genotype 1) is primarily transmitted through contaminated water [33]. The differences in HEV seropositivity among the subpopulations in our study were likely influenced by exposure to various HEV genotypes and differing transmission routes, such as those encountered during travel to the country of origin.

Although trichinellosis is a rare disease, with only one case reported in Berlin between 2007 and 2016, we detected anti-*Trichinella* antibodies (IgG or IgM) in 2 of the 195 Vietnamese participants. Studies from Vietnamese provinces where *T. spiralis* circulates in pigs have reported a human seroprevalence of 1.6–3.5% [34], suggesting a higher risk of exposure. This highlights the need for increased awareness of trichinellosis among the Vietnamese migrant population in Germany, as well as in travellers returning from Vietnam and other endemic regions.

Acute human brucellosis is a rare disease in non-endemic countries such as Germany, with only four cases reported in Berlin in 2016. Because we did not detect anti-*Brucella* IgG antibodies in the collected blood samples, the overall risk of exposure appeared to be low in the subpopulations studied, even among Turks, despite an incidence rate approximately 30 times higher in this group than in the German population [11]. A larger sample size would be necessary to obtain more accurate seroprevalence estimates of anti-*Brucella* IgG. Future serosurveys on human brucellosis in Germany should focus on populations at risk, particularly those returning from or originating in highly endemic countries such as Syria and Türkiye.

A limitation of our study is that the migrant subpopulations, especially Russian and Vietnamese participants, had smaller sample sizes than intended. The large number and diversity of sampling locations (Table 1) enhanced the representativeness of the sample. However, the smaller subpopulations resulted in less precise estimates, as reflected by wider confidence intervals. Some associations may not have been detected due to limited statistical power. Additionally, convenience sampling may have introduced selection bias, and the cross-sectional design of this study does not allow for causal inferences. Environmental exposures (e.g., contaminated water) and community-level factors (e.g., dietary traditions or sanitation infrastructure) were not assessed and may have contributed to differences in seroprevalence. Moreover, the subpopulations studied were heterogeneous; for example, participants with a Russian background included emigrants from Russia whose ancestors were ethnic Germans. Because of the small sample sizes, we were unable to further stratify potential ethnic subgroups.

Our results provide valuable baseline data on six relevant FBZ in migrant populations that are commonly underrepresented in seroprevalence studies, as well as in the population without a migration background in Germany. The strongest associations between potential risk factors, particularly the consumption of raw pork products, and yersiniosis were observed in the non-migrant (German) population. To enhance risk communication on foodborne illnesses among target groups, migrant-sensitive prevention strategies must take into account language requirements and socio-cultural practices [35]. Educating consumers about the risks associated with consuming raw pork can help reduce cases of yersiniosis and other pork-related foodborne infections, such as HEV and trichinellosis. Future studies should explore whether the observed seroprevalence differences among migrant populations also correspond to variations in the number of clinical cases. This approach will support the development of prevention and control measures tailored to populations at risk.

## Figures and Tables

**Table 1:** Numbers of participants recruited at different sampling locations (n = number of sites) in Berlin from 2014 through 2016.

Location	n	Germans	Russians	Turks	Vietnamese	Total
Clinics (Charité *, Vivantes)	2	298	22	69	7	396
Healthcare centres	4	14	31	66	0	111
Occupational Health Centre of Charité	1	109	3	2	1	115
Migrant associations	35	19	157	116	106	398
Ethnic shops	4	3	0	9	54	66
Festivals	3	17	1	4	7	29
Other ^§^	5	37	1	7	20	65
Total	55	497	215	273	195	1180

* Campus Benjamin Franklin and Campus Virchow-Klinikum; ^§^ Other: restaurants, nursing schools, research institutes.

**Table 2:** Demographic characteristics of the four subpopulations recruited in this study.

	Germans n = 497	%	Russians n = 215	%	Turks n = 273	%	Vietnamese n = 195	%
Sex (female)	306	61.6	175	81.4	194	71.1	136	69.7
Age in years (median, IQR)	46 (31–60)		58 (43–65)		46 (36–53)		44 (29–54)	
Generation of migration								
First (born abroad)	-		215	100	209	77	186	95
Second	-		0	0	64	23	9	5
Age in years at migration to Germany (median, IQR)	-		42 (31–51)		18 (4–24)		25 (21–31)	

**Table 3:** Multivariable logistic regression analyses for *Campylobacter*, *Salmonella*, *Yersinia*, and hepatitis E virus (HEV) seropositivity.

Exposure Risks		*Campylobacter*		*Salmonella*			*Yersinia*			Hepatitis E Virus		
	OR	95% CI	*p*-Value	OR	95% CI	*p*-Value	OR	95% CI	*p*-Value	OR	95% CI	*p*-Value
**Migration background (Germans as reference group)**												
Russian				1.6	1.1–2.3	0.008	0.4	0.2–0.6	<0.001	0.3	0.2–0.4	<0.001
Turkish				0.6	0.4–0.9	0.008	0.5	0.3–0.8	0.001	0.6	0.2–0.9	0.01
Vietnamese				2.3	1.5–3.2	<0.001	0.3	0.2–0.4	<0.001	1.9	1.2–2.9	0.005
**Age (18** **–** **29 years as reference group)**												
30–39	0.6	0.4–1.01	0.05				2.1	1.3–3.4	0.002	1.8	0.9–3.6	0.1
40–49	0.7	0.4–1.1	0.1				3.3	2.1–5.2	<0.001	2.3	1.2–4.5	0.01
50–59	0.4	0.3–0.7	0.001				3.2	2.1–5.1	<0.001	4.9	2.7–9.0	<0.001
≥60	0.7	0.5–1.1	0.1				3.5	2.2–5.4	<0.001	10.1	1.2–2.9	<0.001
**Sex (female as reference group)**												
Male				1.3	1.01–1.8	0.045						
**Buying meat products in German retail shops**												
Yes							1.5	1.03–2.0	0.03			
**Consumption of chicken (no consumption as reference group)**												
Rarely				0.7	0.4–1.3	0.3						
Occasionally				0.6	0.3–0.9	0.03						
Often				0.6	0.3–0.9	0.02						
**Consumption of raw pork (no consumption as reference group)**												
Rarely							1.5	0.99–2.2	0.06			
Occasionally							2.6	1.6–4.3	<0.001			
Often							3.4	1.6–7.2	0.001			
**Consumption of raw fish (no consumption as reference group)**												
Rarely	1.6	1.1–2.3	0.01									
Occasionally	1.6	1.0–2.4	0.048									
Often	1.3	0.6–2.9	0.6									
**Careful when buying animal food products**												
Yes							0.6	0.5–0.8	0.001			

Explanatory variables with a *p*-value of <0.05 in the univariable analyses were included in the multivariable models, followed by backward elimination of non-significant variables. Only variables from the final models are presented. Serological test results were considered only when the corresponding questionnaires were completed correctly and in full.

## Data Availability

Data will be made available from the corresponding author on reasonable request.

## Abbreviations

The following abbreviations are used in this manuscript:

CI	Confidence interval
EEA	EU/European Economic Area
ELISA	Enzyme-linked immunosorbent assay
FBZ	Foodborne zoonoses
HEV	Hepatitis E virus
OR	Odds ratio
